# The prevalence of PI^∗^S and PI^∗^Z SERPINA1 alleles in healthy individuals and COPD patients in Saudi Arabia: A case–control study

**DOI:** 10.1097/MD.0000000000008320

**Published:** 2017-10-20

**Authors:** Noura Al-Jameil, Amina A. Hassan, Rana Hassanato, Sree R. Isac, Maram Al Otaiby, Fadwa Al-Shareef, Basmah Al-Maarik, Iman Al Ajeyan, Khloud Al-Bahloul, Samina Ghani, Dana Al-Torbak

**Affiliations:** a Collage of Applied Medical Sciences, King Saud University; b King Khalid University Hospital, Riyadh, Saudi Arabia.

**Keywords:** alpha-1 antitrypsin, genotyping, PI∗S, PI∗Z

## Abstract

Alpha-1 antitrypsin (AAT) is an acute phase protein produced in hepatocytes. Its deficiency affects the lungs and liver. A case–control study was carried out to determine the prevalence of 2 common deficiency alleles, PI∗S and PI∗Z, for alpha-1 antitrypsin deficiency (AATD) in both healthy and chronic obstructive pulmmonary disease (COPD)-affected Saudi populations and to clarify the importance of genetic tests in the screening of people at risk for COPD.

One thousand blood samples from healthy individuals and 1000 from COPD-affected Saudi individuals were genotyped for the above-mentioned alleles, using real-time polymerase chain reaction (PCR), with the exclusion of any other nationalities. Data were analyzed by determining the allele and genotype frequencies through gene counting and its confidence intervals. The allele frequencies, derived by the Hardy–Weinberg equilibrium method, were analyzed by Pearson Chi-squared tests. The confidence intervals for genotype frequencies were calculated using exploratory software for confidence intervals.

Of the 1000 COPD patients included in our study, the prevalence of PI∗S and PI∗Z was 21.8% and 7.7%, respectively, while within the 1000 normal samples, these alleles occurred in 8.9% of patients for PI∗S and 1.6% for PI∗Z. The AAT deficiency genotype frequencies (PI∗ZZ, PI∗SS, and PI∗SZ) were 6.5 per 1000 and 87 per 1000 for normal and COPD-affected Saudi individuals.

Our results indicated a high prevalence of AATD alleles in the normal Saudi population and an association between AAT deficiency and pulmonary disease development. Additionally, our research confirms the importance of genetic screening to achieve early and accurate diagnosis of AATD.

## 1 Introduction

Alpha-1 antitrypsin deficiency (AATD) is a common genetic disease that affects the lungs and liver.^[1]^ Alpha-1 antitrypsin (AAT) is a protease inhibitor (PI) that primarily functions in the protection of lungs from neutrophil elastase,^[2]^ which causes destruction of pulmonary parenchyma. The correct expression of the PI locus is essential for the protection of the lungs. AAT is expressed mainly in liver cells (hepatocytes) and is then released into the plasma, where it must be maintained at sufficiently high levels to protect lung elastin from degradation by neutrophil elastase.^[3]^ AATD leads to decreased plasma and alveolar concentrations of this protein, correlating with early emphysema and chronic obstructive pulmonary disease (COPD).^[4]^ Accumulation of abnormal AAT in hepatocytes is also responsible for liver disease.^[5]^

The *AAT* gene is highly pleomorphic with approximately 100 identified alleles. Variants that cause an increased risk for manifesting diseases are those in which the serum AAT concentrations are less than 60%. PI∗M is the most common and physiologically normal allele, while PI∗Z and PI∗S are the most common mutations that produce clinically deficiency of AAT.^[6]^ PI∗ZZ is considered the most pathologic genotype related to AATD, followed by PI∗SZ. Among smokers, PI∗SZ patients are less susceptible to cigarette smoke than PI∗ZZ patients, and the pattern of emphysema may be similar at diagnosis to typical COPD patients.^[7]^ This risk for COPD, also found with the PI∗MZ genotype, appears with advanced age and is affected by several factors including genetics, smoking status, and certain environmental factors.

AATD represents one of the most common genetic disorders in Europe. Its prevalence differs clearly from one country to another and even within the same country regions.^[8]^ From a public health perspective, the prevalence of AATD is essential to know in any community.^[5]^

Saudi Arabia is an Asian country inhabited by different nationalities. Several authors have reported that the highest frequencies of ZZ phenotypes in 20 Asian countries were in Afghanistan, Pakistan, Saudi Arabia and, Thailand.^[9]^ Another study indicated that heterozygous genotypes for Z and S mutations and SZ genotypes were 2.53%, 11.39%, and 3.8%, respectively, whereas homozygous SS genotypes were present in 1.9% of individuals, and no ZZ phenotype was observed when genotyping was done for 158 healthy Saudi individuals.^[10]^

The purpose of our work was to provide an obvious picture about the frequencies of the PI^∗^S and PI^∗^Z alleles, as well as those of deficiency genotypes (ZZ, SS, and SZ) in healthy individuals and COPD patients in Saudi Arabia. By collecting many samples as possible as from all over the Kingdom to proof that AATD is not only a disease of Caucasians (or whites) but also affects in many different races such as Saudi people, and to clarify the importance of genetic diagnosis in the screening of people at risk for COPD.

## 2 Methods

### 2.1 Sample collection

A case–control study was designed through collaboration with hospitals in different regions of Saudi Arabia that spanned all provinces of the kingdom (North: Tabuk, Al-Jawf, West: Madinah, MakkahJiddah, Central region: Ha’il, Al Qasim, Riyadh, South: Asir, Najran, Jizan, and East: Dammam, Al-Hufuf. This work lasted 2 years from January 2015 to December 2016, while the collection of samples continued from January 2015 to December 2015.

From these regions, we sought to collect blood samples and medical profiles of people belonging to 2 groups. Group 1 was comprised of 1000 patients who had been clinically diagnosed with COPD where their forced expiratory volume in the first second/forced vital capacity (FEV1/FVC) ratio was <70% based on postbronchodilator FEV1 lung function tests.^[11]^ Spirometry was performed according to the American Thoracic Society/European Respiratory Society 2005 guidelines,^[12]^ with ages ranging from 45 to 75. Group 2 consisted of 1000 healthy control individuals were chosen from the same provinces of patients and belonging to both sexes, with ages ranging from 18 to 45 to highlight the importance of early genetic screening in people at risk before the onset of COPD symptoms, as the average age at diagnosis is approximately 45 years. Samples were collected from the 2 groups after obtaining informed consent. This research was approved by the local Institutional Review Board. Table 1 shows the sample cohorts and selection criteria.

**Table 1:**
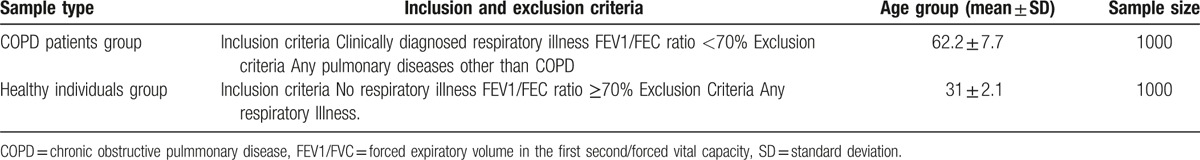
Inclusion and exclusion criteria of samples.

Several basic characteristics, including number of participants, AAT concentration, age, smoking history, and FEV1/FVC, were recorded. Descriptive analysis of these variables was performed; categorical variables were presented as numbers and percentages and continuous variables as the mean and standard deviation (Table 2).

**Table 2:**
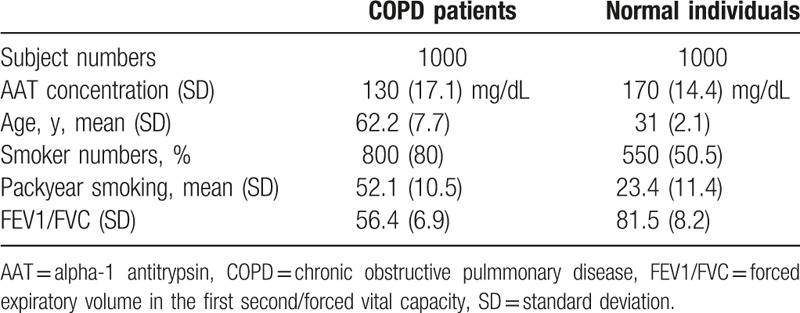
Basline characteristics for patients and healthy individual included in the study.

Tobacco intake was determined as smoking pack years based on the smoking style, daily consumption and total years smoked. Twenty cigarettes smoked per day for 1 year counted as 1 pack year; for nonsmokers, the tobacco intake was defined as 0.

AAT levels were calculated by Nephelometry using BN prospect Immune Nephelometry (Siemens Healthcare, Malvern, PA). The test was carried out as per the instructions in the manual, with a normal reference range from 120 to 200 mg/dL.

The prevalence of deficiency alleles PI^∗^S and PI^∗^Z in the Saudi community was determined by collecting blood samples from patients and normal Saudi individuals, excluding all other nationalities. These 2 alleles were genotyped by real-time polymerase chain reaction (PCR) for rapid, reliable, and inexpensive genotyping, as proven in a previous paper from our project.^[13]^

### 2.2 Deoxyribonucleic acid (DNA) extraction and real-time fluorescence assay

DNA extraction was carried out using a QIAamp DNA Mini kit and a Blood Mini kit from the Qiagen Company, according to the manufacturer's instructions. Real-time PCR was used for genotyping as per Kaczor et al 2007.^[14]^ A pair of PCR primers and a pair of dual-labeled allele-specific fluorescent probes were used (4 oligonucleotides). Synthesis of primers and probes was carried out by the Qiagen Company with sequences as per.^[14]^ (Table 3).

**Table 3:**
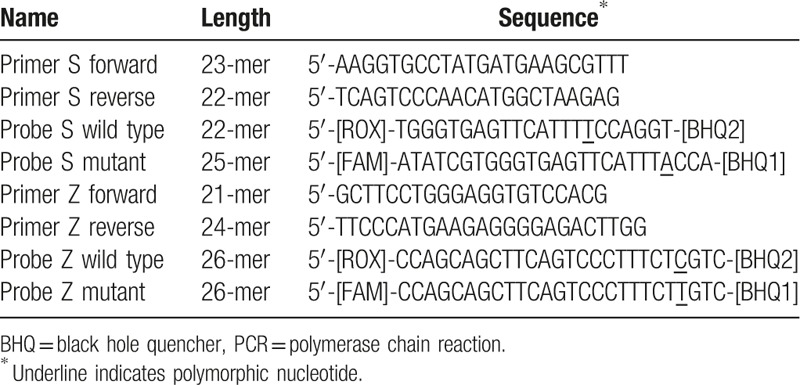
Primers and probes sequences for real-time qualitative PCR assay, as per Kaczor et al, 2007^[14]^.

Complementary to the flanking sequence of the mutations, 2 sets of probes were labeled at the 5-prime end with a reporter dye and at the 3-prime end with the quencher (Black Hole Quencher). The probes differed only by the 1 variable nucleotide for the PI∗Z allele reaction. The probes for a wild-type sequence in the PI∗S reaction are shifted by 3 nucleotides upstream and shortened by 3 bases to enhance the specificity of the assay. The variable nucleotides for each of the sets of probes were near the 3 prime ends. Probes for mutant alleles were labeled with 6-carboxyfluorescein, while those for the wild-type alleles were labeled with 6-carboxy-X-rhodamine (ROX).

The allelic discrimination assay was based on 5-prime nuclease activity of *Taq* polymerase and was carried out in a total volume of 50 μL containing 3 μL of the genomic DNA template, 20 pmol of each primer (TIBMOLBIOL), 1 U of *Taq* polymerase (Finnzymes), 2 pmol of each probe (IDT DNA, Coralville, IA), 10 nmol of each dNTP (Fermentas), and 2% dimethyl sulfoxide (Sigma, St. Louis, MO) in a buffer composed of 15 mmol/L ammonium sulfate, 60 mmol/L Tris HCL, pH 8.9, 3.5 mmol/L magnesium chloride, 0.02% Tween 80, and 0.002% mercapto-ethanol.

A thermocycler was used for amplification with an optical module (iCycleriQ; Bio-Rad, Hercules, CA). In each cycle, fluorescence data were collected at the end of annealing/extension using appropriate filter sets: excitation/emission for ROX at 575/620 nm and for 6-carboxyfluorescein at 490/530 nm. Analysis of genotyping results was determined with iCycleriQ Optical System Software v.3 (Bio-Rad).

### 2.3 Statistical analysis

Data were analyzed by determining the allele and genotype frequencies through gene counting and its confidence intervals. The expected numbers of AAT genotypes in the Saudi population were extrapolated through observed genotype frequencies in COPD patients and normal individuals. The prevalence of each genotype in the population was obtained by dividing the number of estimated genotypes by the total population number. The allele frequencies, which were derived by the Hardy–Weinberg equilibrium (HW) method for COPD patients and normal individuals, were analyzed by Pearson Chi-squared tests. The confidence intervals for genotype frequencies were calculated using exploratory software for confidence intervals (ESCI JSMS, La Probe University and Melbourne, Australia).

## 3 Results

**Figure 1:**
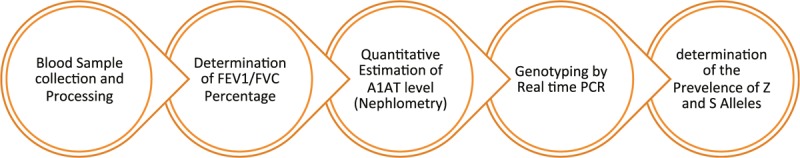
Subjects flow chart.

At the beginning of our work, 1150 samples were collected from individuals with COPD. All patients who participated in each stage of the research had full data, except for 150 samples, which were excluded for different reasons including no informed consent (75), insufficient amount of sample material or poor quality (65), and incorrect COPD diagnosis,^[10]^ while for the control number, was 1000 at the beginning and the end of the study. Figure 1 represents the various steps of each patient's diagnosis.

A total of 2000 blood samples and medical profiles were collected from people in the 13 provinces around the Kingdom, belonging to 2 groups. Group 1 was comprised of 1000 patients who had been clinically diagnosed with COPD, with ages ranging from 45 to 75. Group 2 consisted of 1000 healthy control individuals were chosen from the same social classes and from the 13 provinces of patients and belonging to both sexes, with ages ranging from 18 to 45.

In a sample of 1000 COPD patients, it was observed that the number of patients homozygous for MM was 600, SS was 107, and ZZ was 20, and the number of patients heterozygous for MS was 160, MZ was 50, and SZ was 63. The HW-observed allele frequencies of these patients were 705 for the M allele, 218 for the S allele, and 77 for the Z allele. Based on these observed frequencies of ATT genotypes of COPD patients, the expected numbers of genotypes were derived for the general Saudi population. The prevalence of these genotypes (MM, MS, MZ, SS, SZ, and ZZ) was found to be in the range of 1.7 to 50. (Tables 4 and 5).

**Table 4:**
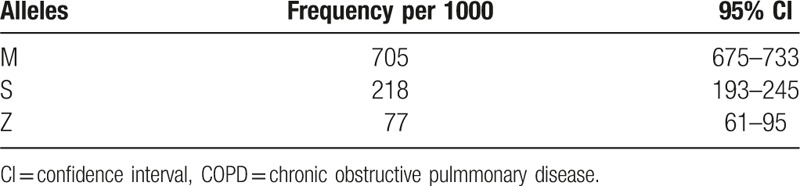
Observed allele frequencies and CIs for COPD patients in Saudi Arabia.

**Table 5:**
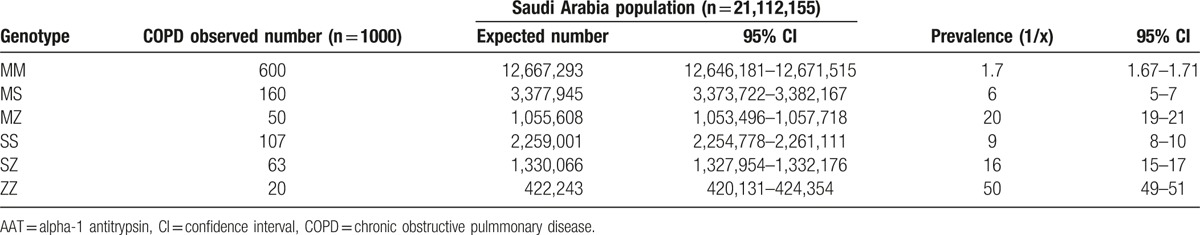
Expected number of AAT genotypes in Saudi Arabia and its CI derived from COPD observed number of patients.

In the sample of 1000 normal Saudi individuals, the distribution of homozygosity for MM was 800, SS was zero, and ZZ was 2, and heterozygous for MS was 170, MZ was 20, and SZ was 8. The HW-observed allele frequencies of these normal individuals were 895 for the M allele, 89 for the S allele, and 16 for the Z allele. By using the observed frequencies of ATT genotypes of normal individuals, the expected number of genotypes was derived for the general Saudi population. The prevalence of these genotypes (MM, MS, MZ, SZ, and ZZ) was found to be in the range of 1.43 to 500, while there was no SS genotype in the general population (Tables 6 and 7).

**Table 6:**
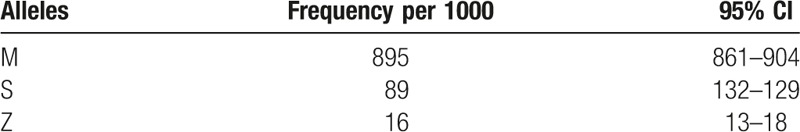
Observed allele frequencies and confidence intervals (CIs) for normal Saudi individuals.

**Table 7:**
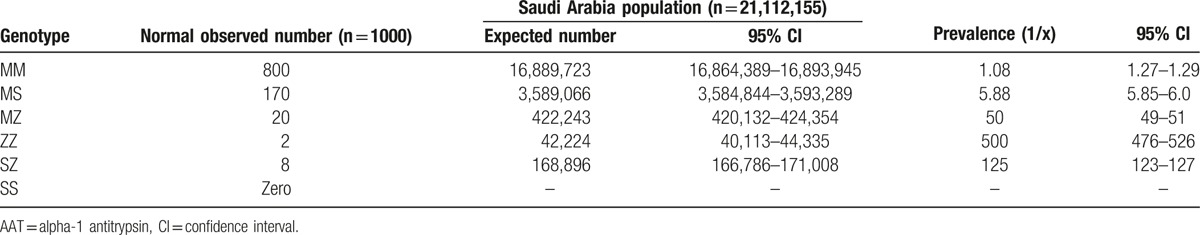
Expected number of AAT genotypes in Saudi Arabia and its CI derived from normal subjects.

There is a highly statistically significant difference in the distribution of allele frequencies (M, S, and Z) between COPD patients and normal individuals (χ^2^ = 94.212, *P* < .001).

The expected genotype HW frequencies for COPD patients and normal Saudi individuals for MM, MS, MZ, ZZ, and SZ and SS are given in (Table 8).

**Table 8:**
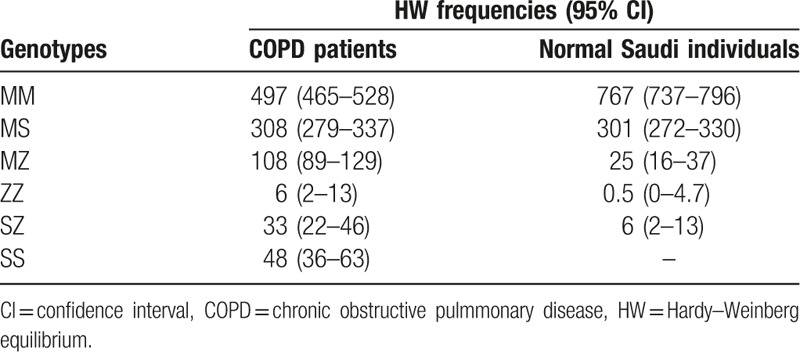
Expected genotypes HW frequencies for COPD patients.

## 4 Discussion

Carriers for metabolic diseases such as AAT deficiency are at risk for adverse health effects and susceptibility to COPD and liver disease as infants as well as adults.^[15,16]^ PI^∗^Z and PI^∗^S are the most commonly mutated alleles that produce clinical deficiency of AAT.^[6]^

Our study demonstrates the prevalence of the above-mentioned alleles in both the healthy and COPD-affected Saudi population. A high prevalence was recorded for PI∗S mutation (15.3%) in normal individuals compared to that in several European areas, including Madeira Island (18%), Portugal (12.9%), and Spain (10.4%).^[17]^ Our PI∗Z mutation showed frequencies of 1.6%, while these frequencies were 2.5%, 2.26%, 2.45%, 2.45%, 2.3%, 4.09%, and 2.57% in the European areas of Madeira Island, Denmark, Estonia, Poland, Sweden, Latvia, and New Zealand, respectively, for normal populations.^[18,19]^

Another study has described that both the PI∗S and PI∗Z alleles are found in 18 out of 20 Asian countries and demonstrates very striking differences for the distribution of the PI∗S and PI∗Z AATD alleles among these Asian countries. These deficiency alleles were not found in Nepal, while the PI∗Z deficiency allele was present in Kazakhstan.^[9]^ The same authors stated that for a total population of 1,572,802,445 individuals from the 20 Asian countries, the estimated number of ZZ people was 7264; the calculated numbers for each of the 20 countries were Pakistan (14,029), Thailand (11,330), Saudi Arabia (5929), Afghanistan (2942), Tajikistan (1670), and South Korea (1478). In contrast, no ZZ individuals were found in Jordan, Nepal, Indonesia, Singapore, China, or the Philippines, and the lowest number was found in Israel (2), followed by Japan (4), Malaysia (42), Kazakhstan (87), Russia (110), India (205), and Iran (518).

This previous study also demonstrated that in Asia there are 46,492 SZ and 6,672,479 MZ phenotypic individuals and approximately 37 million individuals with MS and SS phenotypes. In a 2009 study carried out in the Madeira Island population, the frequency of all AATD genotypes (ZZ, SZ, and SS) was recorded as the highest in the world (40.9) (95% CI: 14–68).^[15]^ Our frequency of the above-mentioned genotypes was 6.5.

The number of individuals who develop clinical disease with PI∗MZ and PI∗SZ phenotypes is clearly lower when compared with PI∗ZZ.^[9]^ Pathology of AATD is mostly linked to the Z allele, and 96% of subjects have a ZZ phenotype, while the remaining 4% are generally related to SZ and MZ, and a minor percentage belong to other rare deficiency or null phenotypes.^[18,20]^ There is no obvious proof of a link between the PI∗SS and PI∗MS phenotypes and AATD-related diseases.^[21–23]^

In the 20 Asian countries, the mean deficiency allele frequencies were 5.4 (95% CI 4.9–6.1) for PI∗S and 2.2 (1.8–2.6) for PI∗Z, while in European countries, the frequencies were 37 (36–38) for PI∗S and 14 (13–14) for PI∗Z.^[19]^

When the phenotypic data taken from 21 countries in Europe were compared with the data of the 20 countries in Asia, significantly higher numbers in each of the 5 phenotypic classes of AATD (ZZ, SS, SZ, MS, and MZ) were found in the 20 Asian countries compared to the 21 countries in Europe. The origin of these deficiency alleles may be a result of the movement of people over time to major cities, for example, in Pakistan, as well as in Saudi Arabia.^[9]^ It is clear that AATD is not just a disease of Caucasians (or whites) but affects many different races throughout the world.^[18]^

In 2002, several authors^[24]^ examined 1060 subjects with COPD in the USA, and the frequencies of PI∗MS and PI∗MZ were reportedly similar to those of the normal population. In another study,^[25]^ the percentage of ZZ prevalence was 3.2% in 969 patients with COPD. In one of the largest studies of COPD patients,^[26]^ 3152 patients were considered eligible for analysis. The prevalence of severe AATD was 0.63%, including ZZ homozygotes and SZ heterozygotes.

Differences have been reported for the prevalence of AATD associated with COPD in various studies. This variation may be due to several factors including sample sizes, selection criteria, screening program methodology, baseline characteristics of the study populations, and laboratory techniques used.

Saudi Arabia is one of the Asian countries where the susceptibility of carriers for PI∗S and PI∗Z did not allow for mass screening because the cohort sample sizes were small and needed to be expanded both in size as well as in geographical location.^[9]^

In the Saudi Kingdom, several previous studies have not covered all the Saudi Arabian provinces, with relatively small sample sizes including that of Warsy et al (1991),^[27]^ who found that the prevalence of SS, MS, ZZ, and MZ genotypes in healthy people was 0%, 9.31%, 0.49%, and 2.45%, respectively. This study was carried out in the central province. Aljarallah et al (2011)^[10]^ investigated 158 healthy individuals in Quassim province and found that the prevalence of SS, MS, ZZ, and MZ genotypes was 1.9%, 11.39%, 0%, and 2.53%, respectively. More effort is needed to cover all the Kingdom provinces. In our research, samples were taken from 13 sites around the Kingdom from 1000 COPD-affected and 1000 healthy persons. This allowed us to obtain a clearer picture of the condition around the Kingdom. Additionally, we used a more accurate technique (real-time PCR) in the genetic diagnosis, whereas in the previous studies, separation and determination of the products of the PCR was achieved by agarose gel electrophoresis. In our study, the prevalence of SS, MS, ZZ, and MZ genotypes in normal individuals was 0%, 17%, 0.2%, and 2%, respectively. The most likely explanation to this result is the influence of the many people that arrived to the Saudia in order to work and get money, where it is a rich country, from many places such as Europe, Asia, and Africa. Many of them decided to stay and their descendants are now integrated and mixed in the population. We suggest that many of them could be AAT deficient or carriers of the mutations. Once integrated with the Saudi population they may have contributed to a higher prevalence of S and Z mutations. Also, this effects genetic background, together with environmental factors may be one of the reason of this high prevalence of deficiency alleles.

The size of the large sample in our research led to know the importance of genetic screening of people at risk, where in the normal group, the AATD genotypes included 2 ZZ, 8 SZ, and 20 MZ genotypes, with normal AAT mean values; where 28 individuals were smokers. This may be the result of any inflammation as AAT is an acute phase protein and smoking causes increase in its level as a result of the inflammatory process in the lungs.^[28]^ Several authors have reported that acute phase reaction mildly increases AAT concentrations in the presence of Z alleles.^[29]^ Another studies^[30,31]^ demonstrated that only 5% to 15% of homozygous individuals have been identified; currently, the diagnostic delay exceeds 5 years, and the average age at diagnosis is approximately 45 years.

These results reflect the importance of genetic screening to achieve early diagnosis of AATD, especially in people at risk, such as smokers and COPD patient relatives, to enable the enforcement of preventative measures in genetic groups at risk, such as avoidance of dust and pollution from the environment or certain occupations and cessation of smoking. Additionally, in our work, we study the prevalence of the deficiency alleles in 1000 COPD subjects, leading to proof that there is a relationship between AATD and the development of COPD.

## 5 Conclusions

**Figure 2:**
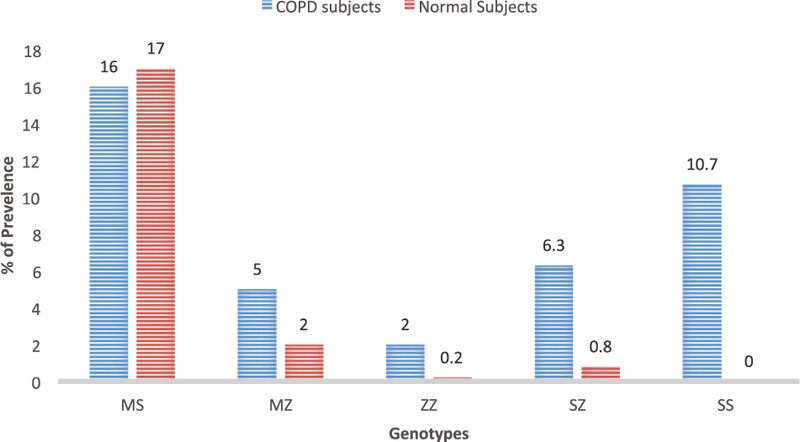
AATD genotypes in Saudi normal and COPD subjects. AATD = alpha-1 antitrypsin deficiency, COPD = chronic obstructive pulmmonary disease.

The prevalence of AATD in the Saudi population is relatively high Fig. 2. The percentage of AATD genotypes in normal Saudi individuals was 17%, 2%, 0.2%, 0.8%, and 0% for MS, MZ, ZZ, SZ, and SS genotypes, respectively. The mean value of serum AAT levels was normal in these individuals, which reflects the importance of valuable genetic test such as real-time PCR for early and accurate diagnosis for individuals at risk and to avoid improper treatment of patients with the condition as the right treatment could save their life. Additionally, the prevalence of the above-mentioned genotypes in subjects with COPD throughout the Saudi Kingdom indicates an association between AATD and the development of pulmonary diseases.

## References

[1] Janciauskiene SM Bals R Koczulla R The discovery of alpha1-antitrypsin and its role in health and disease. Respir Med 2011;105:1129–39. 21367592 10.1016/j.rmed.2011.02.002

[2] Lee WL Downey GP Leukocyte elastase: physiological functions and role in acute lung injury. Am J Respir Crit Care Med 2001;164:896–904. 11549552 10.1164/ajrccm.164.5.2103040

[3] Neto LESM Junior CTD Cardoso GP Pulmonary aspects in alpha1-antitrypsin deficiency. Rev Port Pneumol 2004;10:145–54. 15202033

[4] Needham M Stockley RA Alpha 1-antitrypsin deficiency. 3: Clinical manifestations and natural history. Thorax 2004;59:441–5. 15115878 10.1136/thx.2003.006510 PMC1746985

[5] American Thoracic Society, European Respiratory Society. American Thoracic Society/European Respiratory Society statement: standards for the diagnosis and management of individuals with alpha-1 antitrypsin deficiency. Am J Respir Crit Care Med 2003;168:818–900. 14522813 10.1164/rccm.168.7.818

[6] Fregonese L Stolk J Hereditary alpha-1-antitrypsin deficiency and its clinical consequences. Orphanet J Rare Dis 2008;3:1–9. 18565211 10.1186/1750-1172-3-16 PMC2441617

[7] Green CE Vayalapra S Hampson JA PiSZ alpha-1 antitrypsin deficiency (AATD): pulmonary phenotype and prognosis relative to PiZZ AATD and PiMM COPD. Thorax 2015;70:939–45. 26141072 10.1136/thoraxjnl-2015-206906

[8] Aboussouan LS Stoller JK Detection of alpha-1 antitrypsin deficiency: a review. Respir Med 2009;103:335–41. 19013782 10.1016/j.rmed.2008.10.006

[9] de Serres FJ Blanco I Fernández-Bustillo E Estimated numbers and prevalence of PI∗S and PI∗Z deficiency alleles of alpha1-antitrypsin deficiency in Asia. Eur Respir J 2006;28:1091–9. 17005586 10.1183/09031936.00029806

[10] Aljarallah B Ali A Dowaidar M Prevalence of (-1-antitrypsin gene mutations in Saudi Arabia. Saudi J Gastroenterol 2011;17:256–60. 21727732 10.4103/1319-3767.82580 PMC3133983

[11] Vogelmeier CF Criner GJ Martinez FJ Global Strategy for the Diagnosis, Management, and Prevention of Chronic Obstructive Lung Disease 2017 Report. GOLD Executive Summary. Am J Respir Crit Care Med 2017;195:557–82. 28128970 10.1164/rccm.201701-0218PP

[12] Miller MR Hankinson J Brusasco V Standardisation of spirometry. Eur Respir J 2005;26:319–38. 16055882 10.1183/09031936.05.00034805

[13] Al-Jameil N Hassan AA Buhairan A Genotyping diagnosis of alpha-1 antitrypsin deficiency in Saudi adults with liver cirrhosis. Medicine (Baltimore) 2017;96:e6071. 28178162 10.1097/MD.0000000000006071 PMC5313019

[14] Kaczor MP Sanak M Szczeklik A Rapid and inexpensive detection of ((1)-antitrypsin deficiency-related alleles S and Z by a real-time polymerase chain reaction suitable for a large-scale population-based screening. J Mol Diagn 2007;9:99–104. 17251342 10.2353/jmoldx.2007.060048 PMC1867421

[15] Blanco LE de Serres FJ Fernańdez-Bustillo E alpha1-Antitrypsin and fibromyalgia: new data in favour of the inflammatory hypothesis of fibromyalgia. Med Hypotheses 2005;64:759–69. 15694694 10.1016/j.mehy.2004.10.005

[16] Blanco I Canto H de Serres FJ Alpha1-antitrypsin replacement therapy controls fibromyalgia symptoms in 2 patients with PI ZZ alpha1-antitrypsin deficiency. J Rheumatol 2004;31:2082–5. 15468381

[17] Spínola C Bruges-Armas J Pereira C Alpha-1-antitrypsin deficiency in Madeira (Portugal): the highest prevalence in the world. Respir Med 2009;103:1498–502. 19450958 10.1016/j.rmed.2009.04.012

[18] de Serres FJ Worldwide racial and ethnic distribution of alpha1-antitrypsin deficiency: summary of an analysis of published genetic epidemiologic surveys. Chest 2002;122:1818–29. 12426287 10.1378/chest.122.5.1818

[19] Blanco I de Serres FJ Fernandez-Bustillo E Estimated numbers and prevalence of PI∗S and PI∗Z alleles of alpha1-antitrypsin deficiency in European countries. Eur Respir J 2006;27:77–84. 16387939 10.1183/09031936.06.00062305

[20] Blanco I Fernández E Bustillo EF Alpha-1-antitrypsin PI phenotypes S and Z in Europe: an analysis of the published surveys. Clin Genet 2001;60:31–41. 11531967 10.1034/j.1399-0004.2001.600105.x

[21] de Serres FJ Blanco I Fernández-Bustillo E Genetic epidemiology of alpha-1 antitrypsin deficiency in North America and Australia/New Zealand: Australia, Canada, New Zealand and the United States of America. Clin Genet 2003;64:382–97. 14616761 10.1034/j.1399-0004.2003.00143.x

[22] de Serres FJ Luisetti M Ferrarotti I Alpha-1 antitrypsin deficiency in Italy: regional differences of the PIS and PIZ deficiency alleles. Monaldi Arch Chest Dis 2005;63:133–41. 16312203 10.4081/monaldi.2005.630

[23] de Serres FJ Alpha-1 antitrypsin deficiency is not a rare disease but a disease that is rarely diagnosed. Environ Health Perspect 2003;111:1851–4. 14654440 10.1289/ehp.6511 PMC1241756

[24] Wencker M Marx A Konietzko N Screening for alpha1-Pi deficiency in patients with lung diseases. Eur Respir J 2002;20:319–24. 12212962 10.1183/09031936.02.02012001

[25] Brantly M Mishra V Zienko L Statewide targeted screening and detection of AAT deficiency. Am J Crit Care Med 2003;167:222.

[26] Rahaghi FF Sandhaus RA Brantly ML The prevalence of alpha-1 antitrypsin deficiency among patients found to have airflow obstruction. COPD 2012;9:352–8. 22506682 10.3109/15412555.2012.669433

[27] Warsy AS El-Hazmi MA Sedrani SH Alpha-1-antitrypsin phenotypes in Saudi Arabia: a study in the central province. Ann Saudi Med 1991;11:159–62. 17588073 10.5144/0256-4947.1991.159

[28] Serapinas D Narbekovas A Juskevicius J Systemic inflammation in COPD in relation to smoking status. Multidiscip Respir Med 2011;6:214–9. 22958407 10.1186/2049-6958-6-4-214 PMC3463080

[29] Perez-Holanda S Blanco I Menendez M Serum concentration of alpha-1 antitrypsin is significantly higher in colorectal cancer patients than in healthy controls. BMC Cancer 2014;14:355. 24886427 10.1186/1471-2407-14-355 PMC4032587

[30] Greulich T Vogelmeier CF Alpha-1-antitrypsin deficiency: increasing awareness and improving diagnosis. Ther Adv Respir Dis 2016;10:72–84. 26341117 10.1177/1753465815602162 PMC5933657

[31] Stoller JK Strange C Schwarz L Detection of alpha-1 antitrypsin deficiency by respiratory therapists: experience with an educational program. Respir Care 2014;59:667–72. 24106322 10.4187/respcare.02817

